# Some Microstructural Aspects of Ductile Fracture of Metals

**DOI:** 10.3390/ma14154321

**Published:** 2021-08-02

**Authors:** Wiktor Wciślik, Robert Pała

**Affiliations:** 1 Faculty of Civil Engineering and Architecture, Kielce University of Technology, 25-314 Kielce, Poland; 2 Faculty of Mechatronics and Mechanical Engineering, Kielce University of Technology, 25-314 Kielce, Poland; rpala@tu.kielce.pl

**Keywords:** fracture mechanics, ductile fracture, material microstructure, void growth, FEM model, material testing

## Abstract

The paper discusses the basic issues of the local approach to ductile fracture of structural metals, with particular emphasis on the failure due to microvoid development. The mechanisms of nucleation of voids around inclusions and precipitates are characterized. The criteria for the nucleation of voids resulting from cracking of the existing particles or their separation from the material matrix are presented. Selected results of experimental studies and Finite Element Method (FEM) simulations on nucleation of voids are discussed. The analytical and numerical models of growth and coalescence of voids are described, indicating the effect of the stress state components on the morphology of voids and the course of the cracking on a microscopic scale.

## 1. Introduction

Many engineering structures in use have reached or exceeded their design service life. In combination with changing operational requirements (e.g., increased loads, influence of environmental factors), design, execution, and operational errors can result in numerous failures. Historically, the most famous case of this type was the cracking of the hulls of Liberty tankers in the 20th century. In the following years, cracks in the structures of industrial tanks, gas pipelines, and others were observed many times. As shown by the research and analyses carried out, one of the causes of the damage was the imperfect method of designing and calculating structures that did not follow the rapid technological progress. Extensive research on the explanation of the causes of failures resulted in the establishment of a new field of science, which is the mechanics of fracture. Furthermore today, with the rapid development of material technologies, the importance of this relatively young science continues to grow.

For about 60 years of development, the fracture mechanic has provided models to predict failure of structural components containing defects. The first solutions were developed on the basis of the linear theory of elasticity, gradually developing them in terms of taking into account plasticity and non-linear phenomena. The so-called global approach has proved to be useful in solving engineering problems in which classic material strength methods are not applicable.

Based on the methods of classical fracture mechanics, many engineering procedures have been developed, among which the PD6493 [1], BS7910 [2], R6 [3], FITNET [4], and SINTAP [5] procedures deserve special mention.

The development of material technology and computational methods, mainly numerical ones, which have been progressing in recent decades, has revealed a number of limitations of conventional methods. Their most common disadvantage is their low versatility, because each case of the geometry of a structural element and a defect requires an individual approach. Thus, these procedures are costly and time-consuming [6].

Local methods, developed since the 1980s, are characterized by much wider possibilities, mainly in combination with FEM analysis. The essence of the local approach is the analysis of phenomena that take place in a small area of crack initiation and development (called the process zone).

This paper discusses the basic aspects related to the local approach to the analysis of ductile fracture of metals, taking into account changes in the microstructure of the material, namely the development of voids.

## 2. Mechanisms of Structural Metals Failure

Two basic mechanisms of metals failure are distinguished, namely cleavage and ductile fracture.

Due to its violent, uncontrolled nature and its consequences, brittle fracture has been the subject of advanced research for many years. The mechanism of brittle failure may take the form of intergranular and transgranular fracture. In most metals, the intercrystalline fracture mechanism is related to the cracking of particles arranged along the grain boundaries. Initiating the fracture process requires breaking the interatomic bonds. The increase in the volume of the resulting void is the result of hydrostatic stresses.

Local stresses, necessary to break the bonds, are characterized by significant values compared to the material strength measured on a macroscopic scale. It follows that the crack initiation takes place around stress concentrators, which are usually geometrical discontinuities at the microscopic level (microvoids, notches, inclusions).

The occurrence of the brittle fracture mechanism in ferritic steels is favored by low ambient temperature and high deformation rates [6]. It should also be emphasized that the process of the brittle fracture largely depends on the microstructural structure of the material (e.g., grain size).

**Figure 1:**
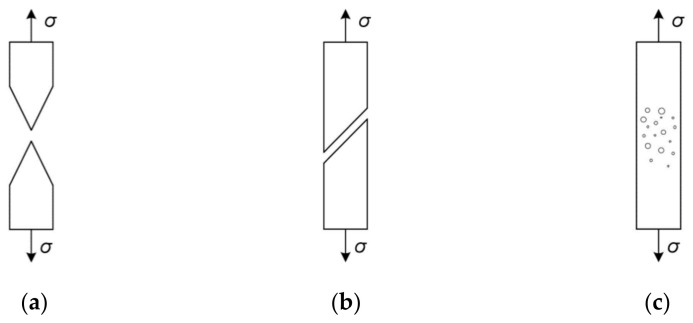
Ductile failure in metals: (**a**) necking; (**b**) shear; (**c**) development of voids.

In typical operating conditions of the structure (static character of loading, room temperature), material failure often takes the form of ductile fracture and is preceded by the occurrence of significant plastic deformation. In metals of high metallurgical purity (copper, gold), in the absence of internal stress concentrators, the failure occurs by necking the cross-section up to a complete narrowing (Figure 1a).

**Figure 2:**
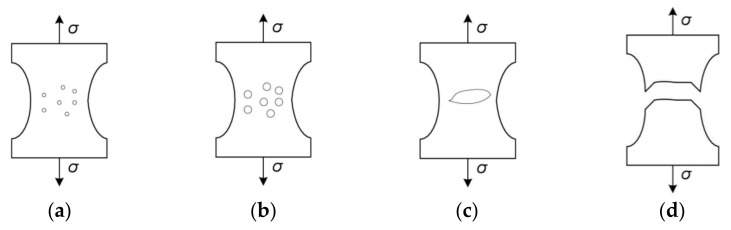
Phases of void development: (**a**) nucleation of voids; (**b**) growth; (**c**) coalescence; (**d**) rupture.

The second process is associated with a slip mechanism in which the shear bands are inclined at an angle of approximately 45 degrees to the axis of the main tensile stresses (Figure 1b). However, ductile fracture in technical metals is most often associated with nucleation and the development of internal microvoids (Figure 1c and Figure 2).

## 3. Some Measures of Stress State and Microstructural Parameters Used for the Ductile Fracture Characterization

As demonstrated in numerous studies, the process of void nucleation and growth in ductile materials is strongly dependent on the stress state. A special role is assigned to a parameter called the stress state triaxiality ratio, which describes the effect of the spherical component of the stress tensor (hydrostatic tension or compression). The stress state triaxiality is defined by the relationship:

(1) $$\eta =\frac{\sigma_{m}}{\sigma_{e}},$$

(the arguments are described in the nomenclature section at the end of the paper).

High values of the triaxiality describe a state in which the effect of hydrostatic stress is significant. In structural elements, such a situation takes place mainly in the vicinity of all kinds of geometric notches, where the local value of η may significantly exceed 2. On the contrary, low triaxiality is observed in structural elements subjected to stress states with a negligible hydrostatic component. For example, uniaxial tension corresponds to the triaxiality value of 1/3.

Many studies, both experimental and theoretical, indicate a significant influence of triaxiality on the intensity of void development, measured by the value of their volume fraction, according to the formula:

(2) $$f=\frac{V_{voids}}{V_{material}},$$

(the arguments are described in the nomenclature section).

It was found [7] that in the case of low triaxiality, due to the minor role of the spherical component of the stress tensor, the void development process is governed by the deviator component, primarily the third stress deviator invariant and the related Lode parameter:

(3) $$L={\left(\frac{r}{q}\right)}^{3}=cos\left(3\theta \right),$$

where: $r=\sqrt[{3}]{\frac{27}{2}\left(\sigma_{1}-\sigma_{m}\right)\left(\sigma_{2}-\sigma_{m}\right)\left(\sigma_{3}-\sigma_{m}\right)}$, $q=\sigma_{e}$.

**Figure 3:**
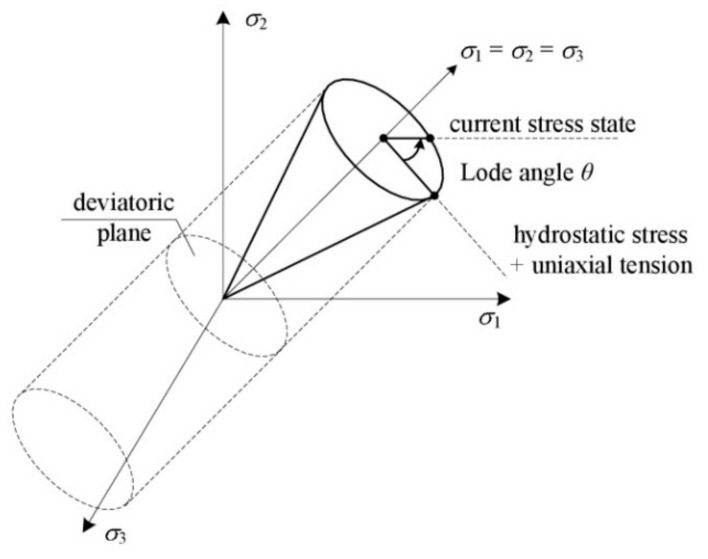
Graphical interpretation of the Lode angle.

The geometric interpretation of the Lode angle in the principal stress space is shown in Figure 3:

The Lode angle satisfies the condition $0\leq \theta \leq \frac{\pi }{3}$, while the Lode parameter is in the range −1≤L≤1.

## 4. Void Nucleation in Metals

**Figure 4:**
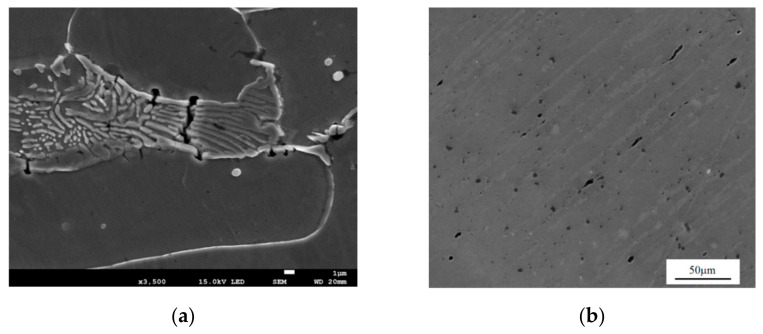
(**a**) Microdamage and voids inside and on the boundaries of the grains; (**b**) an example of impurities in the microstructure of S355 steel.

The fracture of metals usually involves nucleation and the development of microdamage, primarily voids. Their initiators are most often inclusions and the second phase particles, located in the structure of the base material inside the grains or at their boundaries [8,9] (Figure 4a). The particles can be introduced into the metal matrix in order to improve the strength properties (e.g., metal matrix composites, TRIP steels), or may be impurities resulting from the technological process (Figure 4b).

Although the particle sizes are usually of the order of micrometers, and their volume fractions in unstrained material do not exceed a few parts per mille, significant values of localized plastic strains and the occurrence of heterogeneity in the deformation field around the particles cause nucleation and then growth of voids. Their volume fraction at failure may reach several dozen percent.

The presence of inclusions may be the result of imperfections in technological processes and contamination of the material with sulfur, manganese, phosphorus, nitrogen, and other compounds. For example, manganese sulfide commonly present in structural steels is plastic, so when subjected to plastic strain, it deforms to form longitudinal bands that cause fracture toughness anisotropy. The presence of other compounds, in turn, can reduce material strength, ductility, toughness, etc.

On the other hand, in many cases, inclusions and precipitates are intentionally introduced into the structure of the material to achieve certain parameters. For example, the addition of sulfur to the so-called free-cutting steels definitely improves their machinability. Another example of the deliberate introduction of second phase particles is the so-called precipitation hardening, used mainly in soft alloys of aluminum, magnesium, or titanium, in order to improve their strength properties. In this case, the particles themselves should be hard, characterized by high density and uniform distribution in the structure of the base material (matrix). It is also required that the precipitates should be at least partially coherent with the surrounding matrix. The introduced particles constitute a barrier to the free movement of dislocation, which in metals, due to the low resistance of the crystal lattice, occurs relatively easily. Limiting the dislocation movement results in an increase in the strength of the material. The precipitation strengthening technology is also used in composites with a metal matrix.

Regardless of the type and function, the particles of the second phase are, as mentioned above, initiators of the nucleation of microvoids which grow and develop into a macroscopic defect.

**Figure 5:**
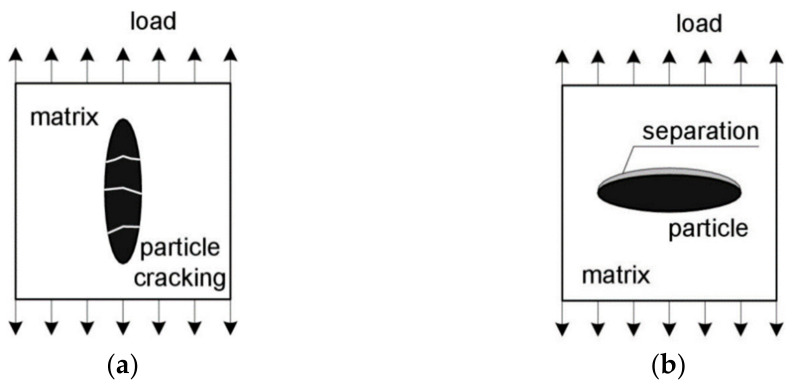
Effect of the orientation of the elongated particle in relation to the loading direction on the void nucleation mechanism: (**a**) the mechanism of fracture of the particle positioned along the loading direction; (**b**) separation of the particle perpendicular to the loading direction, based on [10].

The void nucleation mechanism itself involves the separation (decohesion) of the second phase particle from the matrix and/or the particle fracture. The particle separation mechanism is primarily observed in relatively soft, ductile matrices, while the hard matrix promotes particle fracture which requires lower strain values. The direction of the crack development is usually perpendicular to the direction of the main tensile stresses. Moreover, in the case of particles with an elongated shape, the void nucleation mechanism depends on their orientation with respect to the loading direction. The positioning of the elongated particle along the direction of loading promotes the phenomenon of its cracking (Figure 5a). Otherwise, the particle and the matrix are usually separated (Figure 5b).

**Figure 6:**
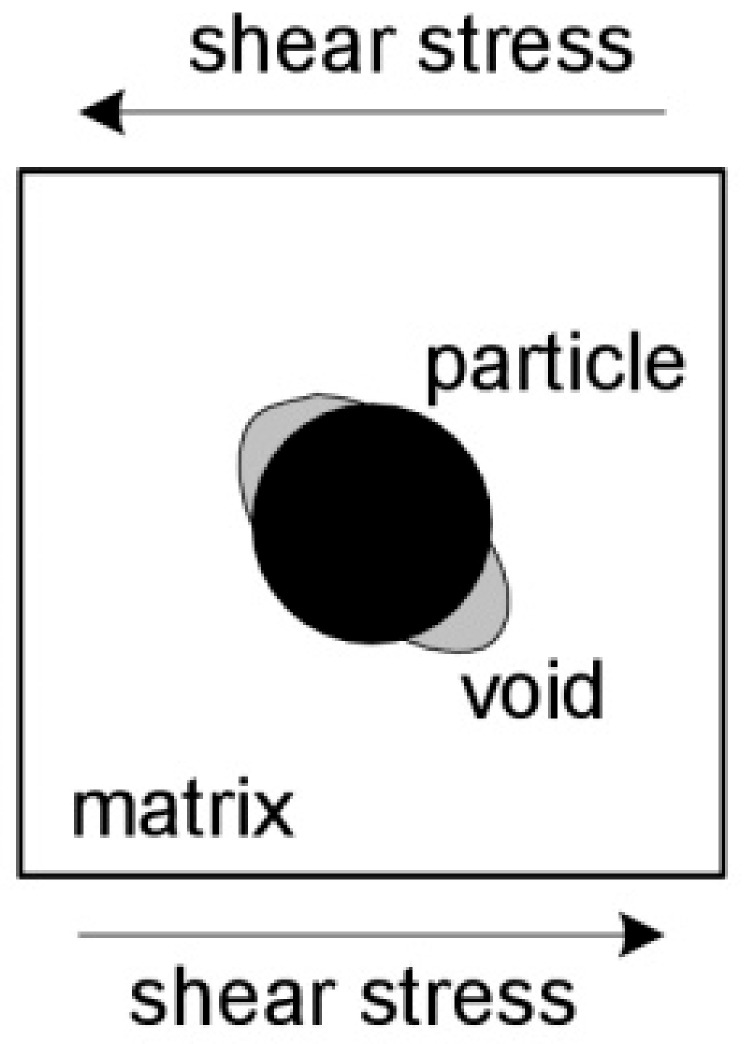
Mechanism of particle–matrix separation under dominant shear stress, based on [11].

Regardless of the factors mentioned above, there is a large group of other parameters that influence the void nucleation mechanism (fracture or particle separation). For example, the phenomenon of particle cracking is favored by the high value of the yield stress, the matrix hardening exponent, high particle stiffness, and the dominance of normal over shear stresses. The phenomenon of cracking is primarily observed in the case of brittle particles, where the failure initiation takes the form of cleavage cracking [11]. In samples subjected to significant shear stresses, the particle–matrix separation at opposite points is observed, as shown in Figure 6.

It should also be remembered that the second phase particle is a stress concentrator on a micro scale, which additionally favors the formation of local discontinuities (voids). A separate issue is the concentration of stress caused by the presence of a group of particles, which also affects the mechanism of microdamage initiation. In this case, the volume fraction of the particles becomes an important parameter. As all authors emphasize, the course of both the nucleation process and the growth of voids is strongly dependent on the spherical component of the stress tensor, the influence of which is described by the so-called stress triaxiality (see Section 3). As it increases, the value of the strain necessary to initiate the void decreases.

The void nucleation process does not take place simultaneously around individual particles, but gradually, depending on the local stress and strain state, the particle material and its size. Microscopic observations of plastically deformed materials show that the nucleation of voids first takes place in the region of larger particles, which, including microdefects, are more susceptible to damage.

In high purity metals (copper, silver, gold), in the absence of second phase particles, the nucleation of microvoids may also result from the accumulation of dislocations [12,13]. Creation of dislocation and slip, although they release shear stresses, do not release normal stresses, which leads to the accumulation of considerable energy. The energy release takes place in the weakest areas, i.e., at the dislocation accumulation points, which leads to the formation of microvoids [14].

Over the last several decades, many criteria for void nucleation have been developed, both on the basis of dislocation theory and continuum plasticity theory. As shown in [15], the scope of applicability of individual criteria is determined primarily by the particle size. The criteria based on the continuum mechanics are used to analyze the separation of particles with a diameter greater than about 1 μm.

The void nucleation criteria can be divided into three basic groups: stress, strain, and energy criteria. The stress criterion, depending on the analyzed void nucleation mechanism (particle fracture or separation from the matrix) requires local achievement of the critical stress value in the particle itself (first mechanism) or at the phase interface (second mechanism), and in its simplest form it is defined by the following relationship (see nomenclature section):

(4) $$\sigma_{1}=min\left(\sigma_{crit}^{particle},\;\sigma_{crit}^{interface}\right),$$

(principal stress $\sigma_{1}$ calculated at microscopic level).

The above formulation, although very simple and convenient, requires knowledge of the principal stresses $\sigma_{1}$ at the microscopic level, and what is more, it does not take into account the particle shape, which undoubtedly affects the conditions of void nucleation. However, the relationship (4) can be modified so that it is possible to determine the local value of stress $\sigma_{1}$ based on the stresses determined on the macro scale, taking into account the particle shape $k_{s}$ [16,17]:

(5) $$\sigma_{1}=\sigma_{1}^{global}+k_{s}\left(\sigma_{e}^{global}-\sigma_{0}\right),$$

(superscript *global* denotes stress measured at macroscopic level).

A thorough analysis of the stress criteria of matrix–particle separation was discussed by Argon et al. in a series of papers [18,19,20]. In the framework of continuum approach, the behavior of the non-deformable particle in an elastic and perfectly plastic matrix was analyzed. The condition of the void nucleation according to [18] relates to the mechanism of the matrix–particle separation and is defined as:

(6) $$\sigma_{m}+\sigma_{e}=\sigma_{crit}^{interface},$$

As the above dependence was derived from the analysis of a single particle in the matrix, it takes into account only the stress and strain state in the particle vicinity, neglecting the influence of the particle size and its possible interaction with neighboring particles. In order to take into account the shape of the particle, the author of [19] proposed a modification of the condition (6) to the form:

(7) $$\sigma_{m}+k_{m}\sigma_{e}=\sigma_{crit}^{interface},$$

It should be noted, however, that the Formulation (7) is only a proposal to solve the problem and has not been supported by experimental research.

The above-mentioned stress conditions assume a homogeneous stress state in the vicinity of the particle, and the distinction between the particle fracture or separation mechanism results only from the adoption of different critical stress values. In fact, in the case of an elastic-plastic matrix, the stress distribution in the elastic particle is not homogeneous.

In [17], attention was drawn to the need to take into account the impact of the heterogeneity of the deformation field in the vicinity of the particle on the separation stress value. Based on the theory of Eshelby [21], a modification of the stress condition was proposed in order to take into account the heterogeneity of the deformation field:

(8) $$\sigma_{1}+\sigma_{inh}=\sigma_{crit},$$

where $\sigma_{inh}$—stress induced in the inclusion by the strain inhomogeneity effect, according to the formula:

(9) $$\sigma_{inh}=\lambda E_{p}\in_{eq},$$

As the authors of the work [17] emphasize, dependence (9) is only an approximate solution. Eshelby theory was developed for an elastic material and its application to a plastically deforming matrix is a considerable simplification. The problem was partially solved by introducing to the description of the plastic material the plastic equivalent Young’s modulus $E_{p}$ and the equivalent Poisson coefficient $\nu_{p}$.

A simple analytical solution for the stress criterion of void nucleation is presented in [22]. The local stress values inside the particle and in the adjacent matrix were determined based on the classic solution of Brown and Clarke [23]. The matrix hardening effect was taken into account by introducing the power equation. The condition proposed in [22] also includes the influence of the volume fraction of particles on the local stress values. The authors’ original achievement was the introduction of the “damage function” Ø into the equation of stresses in a particle, capturing the effect of the separation advancement on the stress values in the particle itself. The particle fracture condition is:

(10) $$\left(\sigma_{0}+k{\left(\varepsilon_{p}\right)}^{n}\right)\frac{\left(1-Ø\right)\left(1-f\right)}{1-f\left(1-Ø\right)}+\frac{1-Ø}{1-f\left(1-Ø\right)}\mu^{*}\varepsilon_{p}=\sigma_{crit}^{particle},$$

where Ø=0 means full traction, Ø=1 denotes complete separation, (the remaining arguments are described in the nomenclature section).

As emphasized by the authors [22], the solution is based on a number of simplifications and can only be applied to some groups of materials. The stress equation does not take into account the mismatch between the elastic parameters of the matrix and the particle. The applicability range of the equation given above is therefore limited to situations where the Young’s modulus of the particle and matrix are of a similar value. Moreover, the assumption of a linear dependence of the “damage function” Ø on the value of plastic deformation is not confirmed by the results of experimental studies. According to the model, the particle loses its stress-carrying capacity only after it is completely detached from the matrix. In fact, it is to be expected that the stress relaxation in the particle occurs somewhat earlier. Finally, the void initiation model presented above does not account for particle size.

Lee and Mear [24] introduced to the nucleation criterion the coefficients taking into account the stress concentration caused by the presence of the particle. Based on the results of numerical calculations, the authors formulated the concept of stress concentration coefficients in the particle itself and on the particle–matrix interface:

(11) $$\kappa^{p}=\frac{\max\left({\left.\sigma_{1}^{p}\right|}_{\delta \leq \beta }\right)}{S},$$

(12) $$\kappa^{I}=\frac{\max\left({\left.\sigma_{\eta \eta }\right|}_{\delta =\beta }\right)}{S}$$

(superscripts *p* and *I* refer to particle and interface, respectively).

The values of the above-mentioned coefficients $\kappa^{p}$ and $\kappa^{I}$ depend on both the global parameters describing the stress state (stress triaxiality), as well as the matrix and particle material parameters (Young’s modulus, yield stress, hardening exponent, Poisson’s ratio) and aspect ratio of the particle.

Taking into account the stress concentration factors, one can modify the stress criterion (4), obtaining the following conditions:

(13) $$\kappa^{I}\sigma_{1}=\sigma_{\mathrm{crit}}^{\mathrm{interface}},$$

(14) $$\kappa^{p}\sigma_{1}=\sigma_{\mathrm{crit}}^{\mathrm{particle}}$$

The void nucleation criterion, taking into account the phenomenon of dislocation accumulation, was formulated in [25]. As in the case of Equations (13) and (14), the nucleation condition is stress-related and combines macroscopic stress values with local effects, using the expression:

(15) $$\sigma_{1}+\sigma_{loc}{\left(r\right)}_{r=r_{1}}=\sigma_{crit},$$

Another nucleation condition, formulated within the dislocation model, can be defined as follows [6]:

(16) $$\mu \sqrt{\frac{\varepsilon_{N}b}{R}}=\sigma_{crit},$$

It should be emphasized that the local stress values, due to their concentration around particles, are higher than the results from the stress analysis on the macroscopic scale [26]. In addition, the distribution of stresses is influenced by the heterogeneous, random distribution of particles.

While the fractured particle is capable of transmitting tensile stress in the direction parallel to the crack, this possibility no longer exists when the matrix is completely separated from the particle. Regardless of the above, fractured or separated particles can transmit compressive stresses.

As mentioned before, a separate category is formed by the strain criteria of void nucleation. Goods and Brown [15] formulated the strain criterion, however, based on the stress values:

(17) $$\varepsilon =\varepsilon_{crit}=1.7\frac{R}{\mu^{2}b}{\left(\frac{\sigma_{crit}^{interface}-\sigma_{m}-\frac{2}{3}\sigma_{0}}{1+2f+0.38\sqrt{f}}\right)}^{2},$$

Another deformation condition was formulated by Hancock and Cowling [27]. Most often, the critical strain condition is achieved at higher external loads than in the case of the critical stress condition.

The energy conditions of void nucleation correspond to the Griffith’s [28] criterion, according to which the value of the energy released during fracture corresponds to the value of energy necessary to create new surfaces (particle fracture surface or the matrix–particle interface). For example, according to the Gurland–Plateau model [29], separation at the interface between the inclusion and the matrix occurs in the elastic range, if the following condition is satisfied:

(18) $$q\sigma =\sqrt{\frac{E\gamma }{R}},$$

Despite numerous studies, so far it has not been possible to define one universal condition that can be applied regardless of the material, state of stress, and others. On the other hand, it is widely recognized that the energetic condition is a necessary but not sufficient condition to create a new void and that the condition of critical stress or strain must be simultaneously satisfied. Thus far, it has not been unequivocally clarified whether the void nucleation is stress or strain controlled. The results presented in the literature provide conflicting evidence on this point. According to the authors of [30], the phenomenon of void nucleation in spheroidized steel depends on the prevailing local stress state. However, studies conducted on aluminum alloys and cast duplex stainless steel [31] indicate the leading role of the strain state.

Assuming a linearly elastic particle material, one can formulate a simple, one-parameter criterion for its failure, and thus for nucleation of the void. Cracking of a particle occurs when the energy release rate exceeds its fracture toughness. A criterion of this type can also be formulated in terms of stress, that is, fracture of the particle will occur if the maximum principal tensile stress inside the particle exceeds the strength of the material. The above criteria are valid for particle radius of the order of 1 µm and above. For smaller particle sizes, criteria for void nucleation are formulated based on dislocation models.

The mechanism of void nucleation by matrix and particle decohesion, due to the occurrence of significant plastic deformation, usually does not allow for the definition of a simple criterion based on a single parameter. In these cases, it is postulated that the criteria of the critical stress at the interface and the energy criterion should be met simultaneously [16].

The strength of the particle–matrix interface depends on the local chemical composition of both phases and is random. There are many works in the literature, attempting to determine the value of the critical stress needed to separate the matrix and particle. One of the first were Argon [19] and Argon and Im [20]. The values of the decohesion stresses of the Fe_3_C particle and the ferritic matrix were determined at the level of 1700 MPa. Similarly, Beremin in [17] determined the critical MnS particle separation stress in A508 steel of about 800 MPa. As noted by the author, the orientation of the elongated particle along the direction of the principal stress implied the particle fracture mechanism (see Figure 5a). The value of the critical fracture stress in this case was about 1100 MPa.

Giovanola et al. [32] determined the value of the critical stress at the interface between the ferritic matrix and carbide inclusions at the level of 3000 MPa. The tests were carried out on samples made of VAR (vacuum arc remelting) steel subjected to various stress states. The experiment included tensile and compression tests combined with torsion. The experiments were interrupted at various stages. Then, the microstructural investigations of the deformed material were conducted, with particular emphasis on the areas where voids were initiated. The state of stress and strain in these regions was determined by means of the FEM, obtaining macroscopic criteria of void nucleation. Stress values in the micro scale were calculated using the dislocation model of Brown and Stobbs [33].

Shabrov et al. [34] linked the results of microscopic examinations of the deformed material with the numerically obtained maps of the stress distribution in order to determine the criteria for the initiation of microdamage in SAE4330 steel. A thorough analysis of the cracking mechanism of brittle titanium nitride (TiN) particles, which are the main initiators of damage development in the tested steel, was performed. The critical value of stress necessary to break the TiN was determined at the level of 2.3–2.4 GPa, with larger particles usually requiring lower stress values.

An example of the use of modern experimental and numerical techniques to determine the nucleation criteria of microdamage is described in [35]. Using the microtomography method, nucleation of voids in commercially pure Al and Al2124 alloy reinforced by spherical hard ceramic particles was observed. The results confirmed the common belief that the type of matrix influences the void nucleation mechanism, i.e., along with the increasing hardness of the matrix, the decohesion mechanism gave way to the particle fracture mechanism. Comparative analysis of void tomographic images with the results of numerical calculations allowed the determination of the critical fracture stress of ZrO_2_/SiO_2_ particles at about 700 MPa and the critical energy density at the level of 2.5 MJ/m^3^. In the case of the particle separation mechanism, it was not possible to clearly establish the separation criterion due to the complexity of the stress and strain state in the particle neighborhood. For the soft matrix (pure aluminum), the critical stress value was estimated at about 250 or 320 MPa for the hydrostatic and normal stress criteria, respectively. Much higher values of both these stresses, of the order of 1000 MPa, were observed in the hard matrix (Al2124 alloy). However, it should be remembered that, according to the authors of [35], the mere achievement of a critical stress value is not sufficient to separate the particle and matrix, but rather a critical combination of stress and strain components must occur.

Exemplary values of local fracture and separation stresses, quoting from [36] are summarized in Table 1.

Significant progress in the field of numerical methods made it possible to perform a multi-parameter analysis of the matrix and particle separation using cohesive models, which usually characterize the interfacial contact using three groups of parameters: maximum stress (normal and shear), separation work Γ, and displacement δ [40,41]. Although the values of cohesive parameters are often treated as material constants, as indicated in [42], their values depend on the stress triaxiality, specimen geometry, and particle size.

In most cases, simulations with the use of cohesive models are limited to the analysis of separation of individual particles or their small groups. However, recently Andersen et al. in [43] have attempted to use a cohesive model to simulate the development of voids in a full-scale plate subjected to tension. In the numerical model, a process zone was distinguished in which the particles were distributed randomly. Analyzing the cases of different amounts and distribution of particles, it was found that the heterogeneity of material properties resulting from the development of voids strongly influences the location of the rupture.

Many studies have attempted to define the value of the critical strain necessary to initiate the void. The commonly accepted value in the literature is $\varepsilon_{N}=0.3$, given by Chu and Needleman [44]. However, determining the critical strain is a much more complex issue and depends on many factors, i.e., material, stress state, particle geometry, and others.

Fisher [45] estimated the value of particle decohesion strain in steels at about 0.6–0.75. On the other hand, in the work of Hahn and Rosenfield [46], a 25–50% share of cracked particles was found in the aluminum alloy deformed by about 0.07.

In the papers [47,48,49,50], using the finite element method, an attempt was made to estimate the critical strain of particle nucleation in structural steels. By analyzing the separation and fracture mechanism of MnS and Fe_3_C particles, the strain value ranging from 0.01 to 0.29 was determined, depending on the adopted nucleation mechanism and the prevailing stress state.

An interesting study on the nucleation of voids through the fracture of silicon particles in aluminum alloys was presented by Caceres and Griffiths in [51]. The authors conducted microstructural investigation of samples made of Al-7% Si0.4% Mg alloys, subjected to tension and bending. The loading was carried out in stages, increasing the plastic strain every 1%. After each phase, microscopic observations of specially prepared surfaces of the samples were performed. Particular attention was paid to the number of nucleated voids, as well as the mechanism of their formation. Alloys differentiated in terms of silicon particle size were tested. While smaller particles detached from the matrix, larger sized particles (especially those with elongated shape) cracked, leading to void initiation. Although it has not been conclusively confirmed experimentally, it is presumed that larger particles contain microdefects, which implies the mechanism of their cracking. Therefore, the crack initiation at the microstructural level was much more rapid in this case and took place at low strains, of the order of 1%.

The experimental tests of CF8M steel samples under uniaxial and complex stress states presented in [52] showed that at the strain of 16%, about 96% of the cells (sub-areas) isolated on the polished surface contain microcracks, which corresponds to a microdamage density of about 100 mm^−2^.

The value of critical void nucleation strain is often determined by means of fitting numerical and experimental results (e.g., force–displacement curve), using a porous material model, such as, for example, the Gurson model [53]. Xia and Cheng [54] thus determined the critical value of the nucleation strain in A533B steel at the level of 0.04. A similar procedure was used by He and co-workers [55] for the analysis of void nucleation in the Al–Al_3_Ti composite, subjected to the complex stress state. The best convergence of the simulation and experiment results was obtained for a much higher value of nucleation strain, amounting to $\varepsilon_{N}=0.5$.

As mentioned above, the void nucleation process is random and depends on many factors, such as the matrix and particle material, particle shape, size, prevailing stress state, etc. Thus, probabilistic approaches are developed in the literature. For example, in [56], the Weibull distribution was used to model the fracture probability of ZrO_2_ particles in an aluminum alloy, depending on strain, particle shape, and volume. The modeling results were in good agreement with the results of microscopic observations.

An approach in which the random nature of the void nucleation process is taken into account by introducing an additional parameter, namely, the nucleation rate, is widely discussed in the literature. When the stress criterion is applied, the nucleation rate is:

(19) $${\dot{f}}_{nucl}=A_{N}\left({\dot{\sigma }}_{1}^{max}+k_{s}{\dot{\sigma }}_{e}\right),$$

where:

(20) $$A_{N}=\frac{f_{0}}{s_{N}\sqrt{2\pi }}exp\left[-\frac{1}{2}{\left(\frac{\sigma_{1}^{max}+k_{s}\left(\sigma_{e}-\sigma_{0}\right)-\sigma_{crit}^{mean}}{s_{N}}\right)}^{2}\right],$$

Within the strain criterion, the nucleation rate function takes the form:

(21) $${\dot{f}}_{nucl}=A_{N}\dot{\varepsilon },$$

where:

(22) $$A_{N}=\frac{f_{0}}{s_{N}\sqrt{2\pi }}exp\left[-\frac{1}{2}\frac{{\left(\varepsilon -\varepsilon_{N}\right)}^{2}}{s_{N}}\right],$$

## 5. Cavity Growth

As already mentioned, the voids grow under increasing plastic strain. In the literature, one can find a number of papers concerning both microscopic observations as well as analytical and numerical models.

The research on void growth was motivated by numerous observations of the microstructure of structural materials subjected to significant plastic deformation. The voids initiated by particle cracking become rounder with increasing strain, while the voids nucleated by the decohesion process gradually lengthen in the direction of the principal tensile stresses. Moreover, the presence of a separated particle inside the void limits the possibility of its contracting perpendicularly to the main loading direction. Moreover, the presence of significant shear stresses promotes the elongation and rotation of the void [16].

Experimental studies involve the observation of the pre-strained material with the use of a scanning microscope and the monitoring of the material microstructure in the process zone by microtomography [57,58,59]. There are also indirect methods of measuring the void fraction, such as the measurement of material density, its stiffness, or electrical resistance [16].

In [59], using the X-ray tomography method, the process of single void growth in the copper matrix was subjected to detailed analysis. In the first stage, rapid growth of the void along the tension direction was observed. The stress concentration caused by the void presence resulted in a strain rate approximately twice as high as the macroscopic strain of the specimen. As plastic strain increased, the increase in the stress triaxiality, due to the formation of the neck, induced a more intense void growth in the direction perpendicular to the loading direction.

There are numerous models of void growth in the literature, describing the increase in the void diameter or the increase in the volume fraction of voids, based on the stress state components and the plastic strain. When analyzing the microstructural aspects of ductile fracture, it should be remembered that the process of existing void growth is constantly accompanied by the nucleation of new microdamage, which complicates the modeling methodology.

One of the first analytical solutions describing a single void growth was formulated by McClintock [60]. The analysis included the growth of a cylindrical void placed in a rigid perfectly plastic matrix, loaded along the longitudinal axis of the cylinder. The McClintock condition determines the change in particle diameter as a function of strain $\varepsilon_{z}$ measured along the axis of the cylinder:

(23) $$\varepsilon_{void}=ln\frac{a}{a_{0}}=\frac{\sqrt{3}}{2}\left|\varepsilon_{z}\right|sinh\frac{\sigma_{m}}{\tau_{0}}-\frac{1}{2}\varepsilon_{z},$$

Additionally, McClintock extended the proposed model to the case of a linearly hardening matrix. According to McClintock, the material ductility increases with the increase in material hardening and decreases with the increase in the precipitates volume fraction and the stress triaxiality.

Rice and Tracey [61] carried out a similar analysis for a single spherical void placed in an incompressible, rigid-plastic matrix. It was further assumed that the dimensions of the void were small compared to the size of the matrix, and the load was applied as a uniform velocity field, away from the void. The law of void evolution can be written as follows:

(24) $$\frac{\dot{R}}{R}=0.283exp\left(\frac{3\sigma_{m}}{2\sigma_{e}}\right){\dot{\varepsilon }}_{e},$$

The value of 0.283 in the above formula was determined by the authors of [61]. In [62], Huang modified this value according to the following relation:

(25) $$0.427\;\mathrm{for}\;\eta >1\;\mathrm{and}\;0.427{\left(\eta \right)}^{1/4}\;\mathrm{for}-\frac{1}{3}\leq \eta \leq 1,$$

where η denotes stress triaxiality (Section 3).

In [63], the influence of the initial void fraction on the value of the coefficient in Formula (24) was also analyzed.

The model proposed by Rice and Tracey is only an estimate of the intensity of void development, as it neglects many factors, such as the effect of void shape changes, void coalescence, secondary nucleation, and others. In later years, numerous modifications of Rice and Tracey’s model were developed, taking into account the shape of the void [64], non-linear hardening and viscosity law [65], and others.

McClintock, as well as Rice and Tracey, also emphasized the significant influence of the spherical component of the stress tensor (negative pressure) on the void growth process, especially the increase in their volume.

In the following years, Thomason [66] analyzed the development of cuboidal voids placed in a rigid perfectly plastic matrix, subjected to hydrostatic pressure and principal stresses $\sigma_{1}$ and $\sigma_{2}$. According to Thomason, below the critical value of the void volume fraction, the failure occurs by necking the specimen cross-section, while in the case of larger values of the void fraction, the decisive factor is the formation of ligaments between voids.

The Brown and Embury model [67] defines the law of void growth as a function of the macroscopic strain. The authors also proposed a criterion according to which void coalescence takes place at the moment when the distance between the voids is equal to their size.

Recently, Sills and Boyce [68], using molecular dynamics (MD) simulations, described a phenomenon in which the growth of voids in an aluminum alloy was the result of dislocation annihilation on the surface of a previously initiated void. The results of the numerical simulation clearly showed that the presence of dislocations in the vicinity of the void significantly accelerates its growth.

Regardless of the solutions discussed above, phenomenological models can be distinguished, in which the evolution of any damage measure is determined as a function of changes in the stress or strain state. For example, the Gurson porous material model [53], modified by Tvergaard [69] and Tvergaard with Needleman [70], assumes the law of increasing the volume fraction of voids according to the formula:

(26) $$\frac{\dot{f}}{f\left(1-f\right)}=\frac{3}{2}q\frac{\sigma_{0}}{\sigma_{e}}sinh\left(\frac{3\sigma_{m}}{2\sigma_{0}}\right){\dot{\varepsilon }}_{e},$$

In the original Gurson condition, the value of the q coefficient was 1; however, in later years its value was shown to be dependent on the stress state, volume fraction of voids, or matrix parameters [71,72].

Benzerga and Besson [73] generalized the above relationship to the case of plastic anisotropy:

(27) $$\frac{\dot{f}}{f\left(1-f\right)}=\frac{3}{h}\frac{\sigma_{0}\sigma_{e}}{\sigma_{h}^{2}}sinh\left(\frac{3\sigma_{m}}{h\sigma_{0}}\right){\dot{\varepsilon }}_{e},$$

In recent years, the issue of material anisotropy caused by the elongated, irregular shape of voids has been particularly intensively analyzed in the literature. The proposed models also take into account both the phenomenon of void rotation and the different sensitivity of elongated voids to stresses in particular directions. The problem of anisotropy is of particular importance in the case of low triaxialities and shear dominance, which results in the location of large deformations and the zone of crack formation.

Gologanu et al. in [74,75,76] generalized the Gurson condition for the case of spheroidal voids. In the first step, the authors [74] analyzed the growth of a prolate spheroidal void in a confocal spheroidal matrix, subjected to an axisymmetric loading. In the next paper [75], the same analysis was performed for an oblate void. In the following paper [76], Gologanu et al. discussed the model of prolate and oblate void growth in the generalized velocity field. The so-called Gologanu–Leblond–Devaux model (GLD model) introduces additional parameters and the laws of their evolution to the original Gurson condition, including the void shape parameter w and additional parameters defining the orientation of the elongated void with respect to the stress axes. Particularly noteworthy is the introduction of the second porosity g parameter into the model, which is of particular importance in a penny-shaped crack (completely flat void). As it is known, in the case of voids of this type, their volume fraction is f=0, which in practice would mean fully dense material and reduction of the Gurson material model to the classical von Mises condition, which is non-physical. The parameter g introduces in these cases the equivalent porosity, equal to the volume fraction of a spherical void with the same radius as the penny-shaped void. The GLD model is also used to develop the criteria for coalescence of voids, especially in the aspect of changing the distance between them, which is the result of an increase in plastic strain.

In the following years, the GLD model underwent numerous developments and modifications. Madou et al. [77,78] provided a general solution for ellipsoidal cells containing confocal ellipsoidal voids, indicating the evolution law of the dimensions of the void along each of the three principal axes. The model proposed by Madou and Leblond also takes into account the rotation of the void around each of these axes.

The problem of the void shape evolution has also been widely discussed by Ponte-Castaneda et al., for example [79,80].

A separate group consists of works in which attempts are made to simultaneously take into account plastic anisotropy and void elongation [81,82]. In these cases, the variables in the constitutive equation are a function of both the anisotropy coefficients h, as well as the volume fraction of voids and their aspect ratio. Moreover, the model described in [81] enables prediction of void closure under pure shear.

As mentioned at the beginning of this section, the presence of the particle inside the void prevents it from tapering perpendicularly to the loading direction. This phenomenon is rarely taken into account in void growth and porous material models. Among the available literature, mention should be made of [83,84].

The process of void growth is also strongly influenced by the effect of their interaction. The problem was analyzed by many authors, including Needleman [85], Tracey [86], and Andersson [87]. In [85], a numerical analysis of the development of cylindrical voids, distributed periodically in two directions, was carried out. The assumption of periodic distribution and symmetry of voids allowed the reduction of the problem to the analysis of a cell with a single, bisymmetric void. The quoted literature allows us to state that the presence of voids clearly affects the value of stress and strain, as well as the plastic strain localization, and thus the material relaxation. The interaction of voids of different sizes has been discussed in [88,89,90].

The paper [88] presents the results of a numerical simulation of the material, in which small-sized voids are arranged regularly, alternating with large ones. With the use of axisymmetric models, the behavior of the material was investigated taking into account triaxialities ranging from 0.3 to 0.9. It was found that localized plastic deformation causes a faster growth of small voids, and that with the increase of triaxiality, the strain values decrease. A significant factor accelerating the development of small voids is the vicinity of larger particles. According to Tvergaard, the influence of large voids is in this case greater than the effects of stress concentration associated with the presence of a crack near a small void.

The mutual influence of particle size and local stress concentrations was investigated by Tvergaard and Niordson [89]. A system of large and small particles arranged alternately was adopted for the simulation. The analysis of voids of various sizes allowed the conclusion that particles with a small diameter, comparable to the characteristic material length, are characterized by a lower growth rate. However, the presence of local stress concentrators (voids, discontinuities) is a factor favoring the development of microdamage. Moreover, it has been shown that the rapid growth of small voids predicted by traditional theories of plasticity does not correspond to the experimental results. The authors investigated the influence of stress triaxiality on the value of the volume fraction of large and small voids.

Faleskog and Shih [91], using the axisymmetric model with parallel, cylindrical voids, proved that the presence of large voids in the vicinity of a blunted crack is a factor that initiates rapid, unstable development of small voids.

In general, the above papers on constitutive modeling and numerical simulation of void growth are only some examples, subjectively selected from the number of publications available. Numerous modifications of the void growth models also take into account strain and kinematic hardening, rate dependency and viscoplasticity, void size, and others. A more extensive review can be found, for example, in [16].

Regardless of constitutive modeling, the use of X-ray microtomography to analyze changes in the material microstructure has contributed to a better understanding of the nature of void growth and coalescence. In [92], Seo et al. described an example of the application of the microtomographic method to a comprehensive analysis of the microdamage evolution in tensile, flat JIS SUM24L steel specimens. The change in the number and volume fraction of voids as a function of plastic deformation was recorded. The tomographic examinations were also used to observe the specimen necking. On the basis of the obtained results, the components of the stress state in the area of void observation were determined.

Contrary to expectations, the average size of the observed voids decreased with increasing plastic deformation. The authors suspect that this is the result of the domination of the secondary nucleation mechanism over the development of the existing voids and the related increase in the proportion of small-sized voids.

## 6. Cavity Coalescence and Failure

The process of void coalescence immediately precedes the formation of the macroscopic defect. This process is currently the least recognized phase of ductile fracture. The occurrence of this phenomenon has been confirmed experimentally [59,93], although these are still few observations [11].

**Figure 7:**
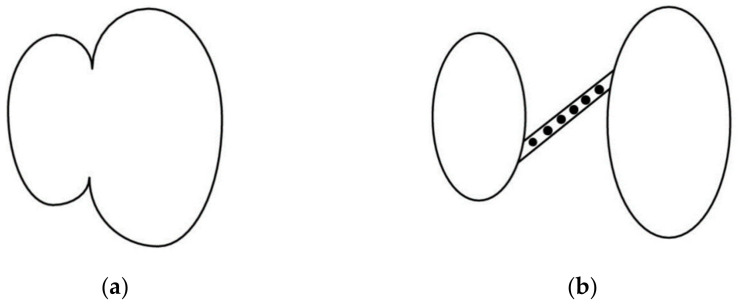
Mechanisms of cavity coalescence: (**a**) internal necking of ligaments between voids; (**b**) nucleation of secondary voids in shear bands [6].

There are two basic mechanisms in metals (Figure 7). In the first one, the coalescence takes place by internal necking of ligaments between voids, similar to the formation of a neck in a tensile specimen. The second mechanism, involving the coalescence of large voids in the shear bands, is most common in high strength metals.

The transition from shear to the necking mechanism is associated with a change in the macroscopic fracture surface, that is, with the transition from the slant to flat fracture [36] (compare Figure 11 in Section 7). The literature also describes a third mechanism related to the coalescence of small voids arranged in strips or joining larger, elongated voids. Each of the mechanisms listed here is divided into several categories, depending on the parameters of the microstructure of the material, stress state, and others. It should also be mentioned that void coalescence can be a process controlled by both nucleation and growth. Generally, this phenomenon is assumed to involve transition from uniform deformation to localized deformation of the ligaments between the voids.

Various analytical models and void coalescence criteria have been described in the literature. It should be noted, however, that in order to fully describe the cracking process, it is not enough to formulate the criterion of the coalescence initiation, but additionally it is required to model the behavior of the ligaments up to failure.

The Brown and Embury [67] void growth model, described in the previous section, also allows for the estimation of critical void coalescence strain:

(28) $$\varepsilon_{coalescence}\approx \frac{1}{C}ln\left[\alpha \left(\frac{1}{\sqrt{f_{0}}}\right)-1\right],$$

As the Brown and Embury model does not take into account the local macroscopic instabilities due to the formation of the neck or the location of the shear bands, it should be treated as an approximate solution.

Thomason presented an analysis of the problem in [64,94]. The author provided the criterion for coalescence of voids in an elastic perfectly plastic matrix. The problem concerned the phenomenon of the location of plastic strains in the ligaments between the voids under the conditions of elastic unloading of the material outside the ligaments. In its original form, Thomason’s criterion is expressed by the equation:

(29) $$\frac{\sigma_{n}}{\sigma_{0}\left(1-\chi^{2}\right)}=\alpha {\left(\frac{1-\chi }{\chi^{W}}\right)}^{2}+1.24\sqrt{\frac{1}{\chi }},$$

According to Thomason’s condition, void coalescence is initiated when the stress normal to the localization plane reaches the critical value of $\sigma_{n}$. Moreover, the influence of the volume fraction of voids on their coalescence is indirectly taken into account by introducing the parameter χ into the model. It should be noted, however, that the Thomason criterion concerns only the initiation of the void joining process, which is not sufficient to model the material in the pre-failure stage. As already mentioned, determination of the evolution law is additionally required in this case. In this respect, the Thomason model was developed by Benzerga [95], who introduced the evolution law of W and χ parameters as a function of plastic strain.

The Thomason criterion in its original form does not take into account the influence of shear stresses. Coalescence models for the case of simultaneous occurrence of tensile and shear stresses were developed in [96,97].

Simple coalescence criteria can also be formulated based on the void growth models described in the previous section. In this case, the achievement of a critical size of voids is decisive for initiating the coalescence. For example, the coalescence criterion based on Rice and Tracey’s void growth law ([61], Section 5) would take the form:

(30) $$\frac{R_{actual}}{R_{0}}={\left(\frac{R_{actual}}{R_{0}}\right)}_{crit},$$

subscript *crit* denotes critical void growth.

An equivalent criterion formulated in [67], as well as in [15], is associated with the achievement of a critical void volume fraction, which was determined by the authors in the range of 0.15–0.25.

The condition of void coalescence adopted in the Gurson–Tvergaard–Needleman (GTN) material model is also based on the critical volume fraction of voids [53,69,70]. The GTN solution includes the definition of the critical void volume fraction $f_{c}$ at which void coalescence is initiated (compare also [98]) and the law of evolution of the void volume fraction after exceeding $f_{c}$:

(31) $$f^{*}=\left\{\begin{array}{c} f\;\mathrm{for}\;f\leq f_{c}\\ f_{c}+\frac{{\bar{f}}_{F}-f_{c}}{f_{F}-f_{c}}\left(f-f_{c}\right)\;\mathrm{for}\;f_{c}<f<f_{F}\\ {\bar{f}}_{F}\;\mathrm{for}\;f\geq f_{F} \end{array}\right.,$$

where:

(32) $${\bar{f}}_{F}=\frac{q_{1}+\sqrt{q_{1}^{2}-q_{3}}}{q_{3}},$$

The influence of various factors on $f_{c}$ has been the subject of many studies. Koplik and Neeedleman [72] showed that it depends mainly on the initial porosity; however, no significant influence of the matrix parameters or stress triaxiality was found.

In [99], the dependence of $f_{c}$ on the initial material porosity $f_{0}$ was described. The value of $f_{c}$ ranged from 0.04 for $f_{0}=0$ to 0.12 for $f_{0}=0.06$.

Generally, a huge number of papers have been devoted to the subject of determining the parameters $f_{c}$ and $f_{F}$ (using experimental and numerical methods) and it is difficult to present a comprehensive review. The values of $f_{c}$ in metal alloys with technical application (mainly steels and aluminum alloys) range from tenths of a percent to about 30%, although usually values of a few percent are assumed [100,101,102]. The values of $f_{F}$ were analyzed, inter alia, in [99,103,104,105], obtaining results ranging from a dozen to nearly 70%.

The experimental methodology for determining $f_{c}$ and $f_{F}$, using the material microstructural analysis has been discussed in [47].

The GTN condition in its original form gives relatively inaccurate results under conditions of significant shear stresses. Hence, in the literature, there are various modifications, taking into account the rapid joining of the voids caused by shear. For example, McVeigh et al. [106] added to the law of void evolution a component accounting for this phenomenon. The law of the evolution of the void volume fraction takes the form:

(33) $$\dot{f}={\dot{f}}_{nucl}+{\dot{f}}_{growth}+{\dot{f}}_{coalescence},$$

However, it should be remembered that formulating the criterion of void coalescence in terms of the value of their critical fraction does not fully solve the problem, as it does not take into account the geometry of the intervoid ligaments or the physical phenomena that occur in them.

Nahshon and Hutchinson [107] introduced into the GTN formulation a component, which takes into account the softening of the ligaments, which is the effect of shear stresses. The description of Nahshon and Hutchinson is phenomenological, and the ligament softening is captured by the additional increase in the proportion of voids.

Relatively widespread in the literature are the criteria for connecting voids based on the critical size of the ligaments between the voids and the crack tip. One of the first solutions of this type was presented by Rice and Johnson [108], who assumed that the onset of coalescence occurs when the length of the ligament is reduced to the length of the void in the direction of loading. Tait and Taplin [109], on the other hand, proposed a criterion according to which the voids are joined when the ratio of the main void axis length to the void spacing reaches a critical value, depending on the type of material. A similar criterion was described by LeRoy et al. [110].

The effect of stress triaxiality as well as the shape and size of the voids on the process of void growth and coalescence was discussed by Richelsen and Tvergaard [99], who analyzed numerically an elastic perfectly plastic material containing small-sized voids. Taking into account the ligament necking mechanism, the authors obtained relatively low critical strain, of the order of 0.3.

Richelsen and Tvergaard also indicated that the occurrence of the ligament necking phenomenon is favored by high values of the matrix hardening exponent, as well as the presence of medium and high triaxiality.

In recent years, Gallican and Hurre [111] proposed an analytical criterion for void coalescence, taking into account the plastic flow in the ligaments between voids. The model is valid under the following assumptions: cylindrical void in a cylindrical unit-cell, axisymmetric loads, orthotropic matrix, satisfying the Hill plasticity condition. Moreover, the authors made extensive validation of the model by comparing the results with the results of numerical simulation of void coalescence, indicating their good agreement.

The above-described solutions for nucleation and development of voids allow for the formulation of material models describing the plastic range of material operation and its ductile fracture.

Void cell simulations are most often conducted for periodically distributed voids [112,113,114]. The obtained results indicate that relative void spacing is the key parameter influencing the course of the void development. In fact, various types of heterogeneity are observed in engineering materials, related to the randomness of the chemical composition of the material, size, shape, distribution of voids, and their orientation in relation to the direction of loading, as well as the distribution and orientation of grains [11]. Therefore, void coalescence is not a homogeneous process that occurs simultaneously in the entire volume of the material, but it is initiated in the areas with significant plastic strain.

The considerable progress made in recent years in the field of numerical methods has allowed for a better understanding of the course of void development. An example of a comprehensive FEM analysis of nucleation, growth, and coalescence of voids was presented by Shakoor et al. in [115]. On the basis of a 3D model of the material with randomly distributed particles, the influence of the void nucleation mechanism (matrix separation and particle fracture, see Section 4 of this work) on the further course of the void growth and coalescence was determined. The simulations carried out for 20% of the particle volume fraction showed that the occurrence of the void nucleation leads to the localization of plastic deformation, which in a further stage favors the local increase of the intensity of void coalescence. Such a wide range of analysis required taking into account large plastic deformation, and thus also advanced FE remeshing techniques.

## 7. Effect of Selected Loading Conditions on Void Development

**Figure 8:**
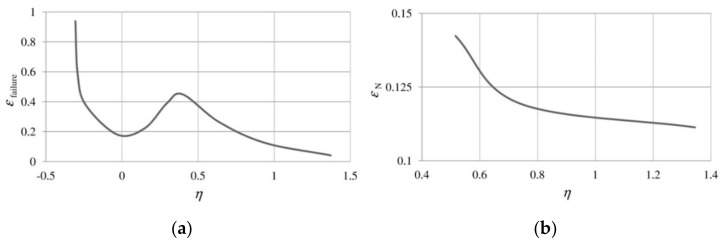
(**a**) Effect of stress triaxiality on strain at failure, from [7]; (**b**) dependence of the mean void nucleation strain on stress triaxiality [47].

The effect of stress triaxiality on void development and ductile failure has been very extensively documented in the literature [63,116]. Moreover, Bao and Wierzbicki [7], examining the 2024-T351 aluminum alloy, showed that the strain at failure is not a monotonic function of triaxiality, but the domain of low and high triaxiality should be distinguished (Figure 8a). In the latter case, an increase in triaxiality corresponds to a decrease in the critical strain.

A similar relationship for medium and high triaxialities was obtained in the already mentioned studies [47,48,49], but in this case, the analysis concerned the void nucleation strain in the S355 structural steel. In the range of triaxialities from 0.516 to 1.345, along with the increase in η, a decrease in $\varepsilon_{N}$ was observed (Figure 8b).

**Figure 9:**
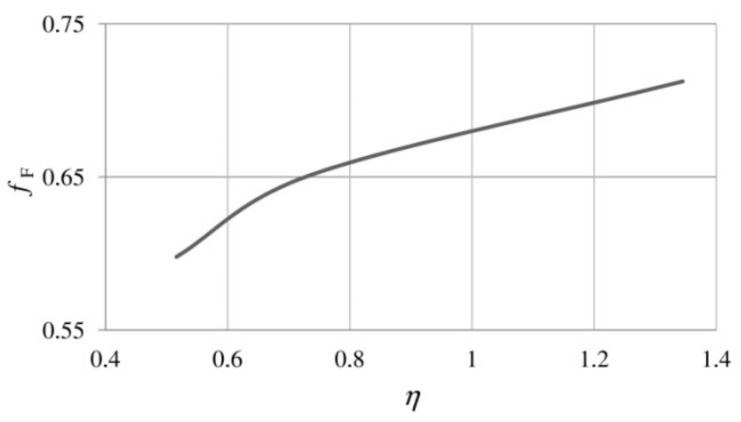
Effect of stress triaxiality on the void volume fraction at failure [47].

High values of triaxiality favor an increase in the volume of voids. Figure 9 presents the experimentally determined (by examining the fracture surfaces) relationship between triaxiality η and the volume fraction of voids at failure in S355J2G3 steel.

Depending on triaxiality, the volume fraction of voids ranged between 59.7 and 71.2%. For comparison, the fraction of voids in the unstrained material was 0.09%.

**Figure 10:**
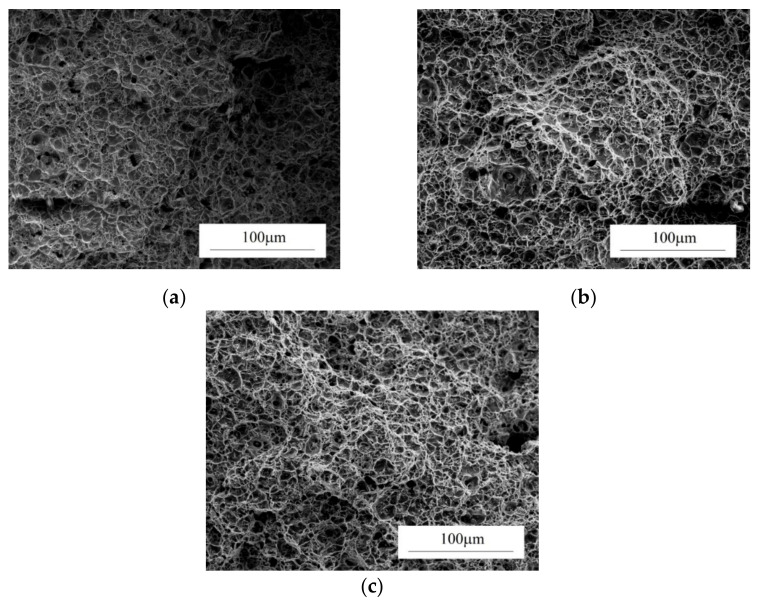
Fracture surfaces of notched tensile specimens made of S355J2G3 steel, subjected to various stress triaxialities: (**a**) 1.345; (**b**) 0.739; (**c**) 0.516.

The stress triaxiality affects not only the intensity of the void growth, but also their shape (Figure 10). In areas with high triaxiality, the voids are spherical in shape (a large share of the stress hydrostatic component forces the voids to grow steadily in all directions—Figure 10a).

As triaxiality decreases (decreasing influence of the spherical component of the stress tensor), the voids become more elongated (Figure 10b,c), because the process of microdamage development in these cases is mainly controlled by shear stresses [117].

As shown by Lin et al. in [118], the reduction of the triaxiality of stresses increases the level of strains at which cracking occurs. For greater triaxial stresses (axis of the specimen), the microvoids grow intensively in the plane perpendicular to the tensile, joining with each other.

Morgeneyer and Besson in [119] presented an example of the successive occurrence of both these mechanisms (regime of high and low triaxiality) in the test of a plate tearing (Kahn test). The observations made by the X-ray microtomography showed that the specimen failure was initiated inside the plate, under conditions of high triaxiality. As the microdamage propagated towards the plate faces, an increasing number of elongated voids was observed, which results from the decrease in triaxiality and the increasing role of shear in the process of void development. It is worth noting that while the shape of the voids undergoes large changes in this case, their volume fraction does not change significantly.

**Figure 11:**
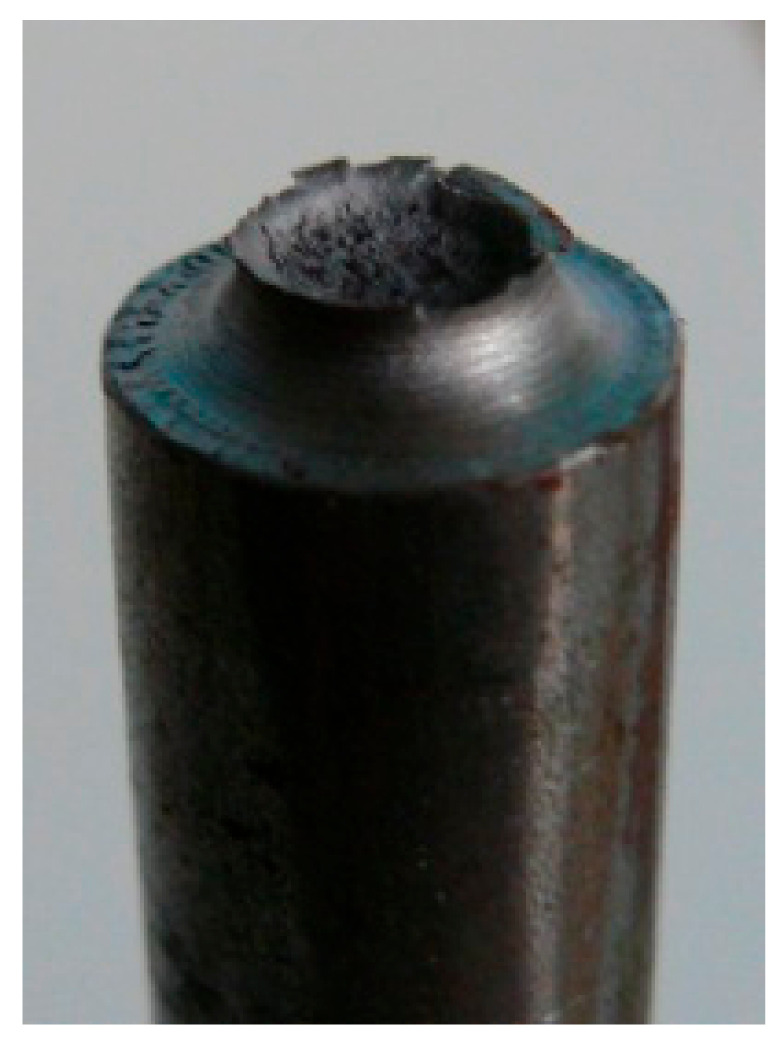
Macroscopic photograph of a fracture surface of a tensile notched specimen. Transition from the flat failure mechanism in the specimen center to the slant fracture at the edges is clearly visible. The first mechanism involves normal stress, while in the latter, shear stress plays a dominant role.

The shear induced failure propagation is also visible on the macroscopic level by the inclination of the fracture surface near the specimen edges (Figure 11).

The development of voids under low triaxiality conditions has been intensively researched in recent years. The small share of the hydrostatic component results in a relatively low increase in the volume of voids. However, in such cases, the action of shear stresses and the associated deformation (change of shape) of the void becomes of primary importance. As it has been shown, in the face of a small value of triaxiality η, the development of voids is in this case are controlled by the value of the Lode parameter ξ (Section 3), although the exact relationships have not yet been defined.

**Figure 12:**
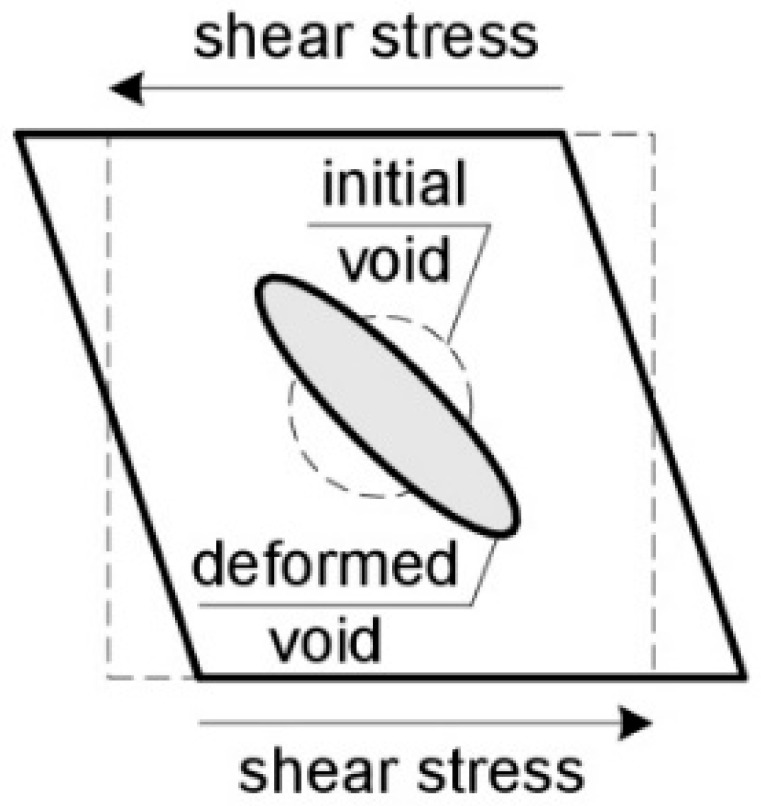
Void deformation and rotation under low triaxiality.

Under shear dominant conditions, voids can take the form of penny-shaped cracks. In addition, the presence of shear stress may cause the voids to rotate (Figure 12), which additionally affects the location of their coalescence area and implies a failure mechanism [11].

In fact, second phase particles still remain inside the void, limiting the possibility of its deformation, especially in the situation of incomplete decohesion at the particle–matrix interface.

Current models of void development primarily take into account the prevailing state of stress, but mostly do not take into account other factors such as deformation rate, temperature, and others, which undoubtedly is of great importance in modeling engineering structures.

Some of the few works taking into account the above factors are [120,121]. It was shown that good results of material description at high temperature are achieved by the use of an Arrhenius phenomenological model [120]. The model takes into account the strain rate for an elevated tensile temperature. The tests were carried out on steel with a bainite structure.

Similarly, the analysis of the influence of very high triaxial strain rates and temperatures in the range of 300–2000 K on the process of nucleation and void development is discussed in [122]. Using the void nucleation and growth model (NAG procedure) as well as molecular dynamics (MD) simulation, the increase in void volume fraction over time was determined, taking into account the process of nucleation, growth, and coalescence. It was found that, while the increase in temperature led to a significant acceleration of the nucleation of the voids, the influence of temperature in the case of the growth of voids was slight.

Another very interesting example of the analysis of the development of voids under dynamic load conditions (spall failure) is [123], in which the phenomenon of stress wave interaction and the associated negative pressure formation, which results in rapid nucleation and void growth, was investigated. Velocimetry on the specimen free surface was used to estimate changes in stress distribution over time. A comparative analysis of the obtained stress distributions and microtomographic photographs of the voids formed made it possible to determine the critical negative pressure necessary to initiate the void, at the level of about 1–2 GPa.

The problem of dynamic development of voids has also been thoroughly discussed in the article [124]. It was found that in the first phase of the failure initiation, the inertia of the material surrounding the void slows down its development, but at a higher value of strain it promotes the void growth. Moreover, in the range of void diameters from 0.1 to 1 μm, the strain gradients around the void contributed to a significant, local increase in the yield point. On the other hand, however, the increase in thermal energy accompanying rapid deformation caused a local increase in temperature even above the melting point, thereby lowering the yield stress, and the stress values necessary to initiate a void, and thus also increasing the intensity of void development.

## 8. Conclusions and Suggestions for Future Research

Due to the practical importance, the issues of microstructural phenomena occurring during ductile fracture of metals have been the subject of intensive research in recent years, which allows for a better phenomenon understanding and the development of advanced computational models.

Although void nucleation has been very extensively documented by the results of experimental tests and simulations, the course of ductile fracture under low triaxiality conditions, i.e., under dominant shear stress, remains an unresolved question. In this case, observations of the fracture surface microstructure indicate the presence of both large and small elongated voids. Their origins have not been fully elucidated so far, although it is believed that such void morphology results from their intensive nucleation at a late stage of deformation, prior to failure, with simultaneous growth of earlier nucleated voids. At present, there are no models of nucleation, growth, and coalescence of voids that take into account the influence of the Lode parameter, which is crucial for the course of ductile fracture under low triaxiality conditions.

As mentioned in Section 6, the stage of void coalescence is presently the least explored phase of ductile fracture. Few papers have been published in which the process of coalescence was extensively investigated in an experimental manner. This is largely due to technical difficulties, although the increasingly widely used method of microtomography seems to be a promising tool. Currently, insufficient understanding of the nature of the void coalescence phenomenon is associated with a small number of analytical models, especially in the case of the simultaneous occurrence of shear and necking of ligaments between the voids. The void coalescence models currently described in the literature are based on certain arbitrarily accepted criteria that have not been sufficiently supported by experimental observations.

A separate problem is the identification of the model parameters. A huge number of papers have been published on this subject, but the dominant approach involves fitting the FEM simulation and the experimental results. However, this approach requires significant experimental and computational effort, and therefore it is inconvenient from a practical, engineering point of view. Moreover, the results obtained in this way apply only to samples with a specific geometry and loading method. Therefore, there is no comprehensive approach that would define standardized parameter values for typical engineering materials.

In conclusion, further research on the development of voids in metals should focus on the following aspects:

- Criteria for void nucleation under low triaxiality conditions;
- Effect of Lode parameter on void initiation, growth, and coalescence;
- In situ observations of void coalescence with the use of modern research methods (e.g., microtomography), which will allow the verification of the existing coalescence criteria, or the development of new ones;
- Assessment of the effect of loading conditions (temperature, strain rate, etc.) on the critical values of stress and strain necessary for the void initiation and growth;
- Development of a set of standardized parameters describing the criteria of nucleation, growth, and coalescence of voids, in relation to engineering materials.

The solution of the above-mentioned problems should lead to the development of practical engineering procedures for estimating the load capacity and safety assessment of structural elements containing material microstructure defects.

## Figures and Tables

**Table 1:** Critical values of void nucleation stresses reported in the literature, from [36].

Particle	Matrix	Critical Stress [MPa]	Ref.
Particle fracture			
Elongated MnS	A508 steel	1100	[17]
Cuboidal TiN	4330 steel	2300	[34]
TiN	Inconel 718	1280–1540	[37]
4% spherical ZrO_2_-SiO_2_	Al2124 (T6)	700	[35]
20% spherical ZrO_2_-SiO_2_	Al2124 (T4)	700	[35]
Particle separation			
MnS	A508 steel	800	[17]
Si	Al (cast)	550	[38]
4% spherical ZrO_2_-SiO_2_	Al2124 (T6)	1060	[35]
4% spherical ZrO_2_-SiO_2_	Pure Al	250	[35]
20% spherical ZrO_2_-SiO_2_	Pure Al	320	[35]
Rounded Fe_3_C	Spheroidized 1045 steel	1650	[20]
Cu-Cr particles	Cu alloy	1000	[20]
TiC	Maraging steel	1820	[20]
C nodules	Cast iron	80	[39]

## Data Availability

No new data were created or analyzed in this study.

## Nomenclature

a	void diameter
$a_{0}$	initial void diameter
b	Burgers vector
f	void volume fraction
$\dot{f}$	the rate of increase in the total fraction of voids
$f^{*}$	actual void volume fraction
$f_{0}$	initial void volume fraction (for unstrained material)
$f_{c}$	critical void volume fraction at the onset of coalescence initiation
${\dot{f}}_{coalescence}$	the rate of increase in the fraction of voids resulting from their rapid joining under shear stresses
$f_{F}$	void volume fraction at failure
${\dot{f}}_{growth}$	the rate of increasing the fraction of existing voids
${\dot{f}}_{nucl}$	the rate of increase in nucleated voids’ volume fraction
h	anisotropy coefficient
k	multiplier in power hardening law
$k_{m}$	coefficient depending on the particle shape
$k_{s}$	coefficient depending on the shape (aspect ratio) of the particle and its orientation in relation to the loading direction
$max\left({\left.\sigma_{1}^{p}\right|}_{\delta \leq \beta }\right)$	maximum value of principal stress in the particle
$max\left({\left.\sigma_{\eta \eta }\right|}_{\delta =\beta }\right)$	maximum value of the stress normal to the phases contact surface
n	exponent in power hardening law
q	stress concentration factor, correction factor in GTN model
$q_{1},\;q_{3}$	Tvergaard coefficients
r	polar coordinate measured from the “head” of dislocation pile-up
$r_{1}$	characteristic length
$s_{N}$	standard deviation
C	coefficient expressing the ratio of the void elongation to the specimen elongation rate (on a macroscopic scale) in Brown and Embury model, C∈1,2
E	Young’s modulus of the matrix
$E_{p}$	matrix plastic equivalent modulus of elasticity
L	Lode parameter
R	particle radius
$\dot{R}$	void radius growth rate
$R_{0}$	initial particle radius
$R_{actual}$	actual particle radius
S	remote normal stress
$V_{material}$	total volume of material
$V_{voids}$	volume of voids and second phase particles
W	void aspect ratio
α	coefficient in Brown–Embury model, coefficient depending on matrix hardening exponent in Thomason model
β	radial coordinate of particle surface in Thomason model
γ	surface energy
δ	generalized radial coordinate in Thomason model
ε	strain
$\dot{\varepsilon }$	strain rate
$\varepsilon_{crit}$	critical strain of particle–matrix separation
${\dot{\varepsilon }}_{e}$	increase in effective plastic strain
$\varepsilon_{N}$	void nucleation strain
$\varepsilon_{p}$	plastic strain
$\varepsilon_{z}$	longitudinal strain of the cylinder in McClintock model
η	stress state triaxiality ratio
θ	Lode angle
$\kappa^{I}$	stress concentration factor at the interface of phases
$\kappa^{p}$	coefficient of normal stress concentration inside the particle
λ	coefficient in Beremin nucleation model, depending on particle shape
μ	shear modulus
$\mu^{*}$	coefficient depending on the elastic parameters of particle, matrix, and the geometric characteristics of particle
σ	stress
$\sigma_{0}$	yield stress
$\sigma_{1},\;\sigma_{2},\;\sigma_{3}$	principal stresses
$\sigma_{1}^{max}$	maximum value of global tensile principal stress
${\dot{\sigma }}_{1}^{max}$	rate of the maximum global principal stress increase
$\sigma_{crit}$	critical stress, dependent on the nucleation mechanism, matrix, particle, and the interface strength
$\sigma_{crit}^{interface}$	critical stress at the phase interface
$\sigma_{crit}^{mean}$	mean value of void nucleation stress
$\sigma_{crit}^{particle}$	theoretical strength of the particle material
$\sigma_{e}$	von Mises equivalent stress
${\dot{\sigma }}_{e}$	rate of von Mises equivalent stress increase
$\sigma_{h}$	Hill’s equivalent stress
$\sigma_{loc}\left(r\right)$	maximum local normal stress at the “head” of dislocation pile-up
$\sigma_{m}$	mean stress (hydrostatic pressure)
$\sigma_{n}$	critical normal stress in Thomason model
$\tau_{0}$	yield stress at pure shear
χ	ratio of void length to the distance between neighboring voids
$\in_{eq}$	equivalent plastic strain
Ø	damage function of the particle–matrix interface
$\nu_{p}$	equivalent Poisson coefficient
